# Supplementary material for: Willingness to pay for an intervention that reduces soda consumption among a sample of middle-class adult Mexicans
Source: PLoS One. 2021 Aug 2;16(8):e0255100. doi: 10.1371/journal.pone.0255100 (PMC8328282; doi:10.1371/journal.pone.0255100)
Supplement: S1 Appendix — (DOCX) [file pone.0255100.s001.docx]

**S1 Appendix. Survey instrument (translated from Spanish to English)**

**Instructions for the interviewer**

**This questionnaire has to be answered only by respondents that reported they had consumed soda in the previous 12 months. If they answered “never”, this questionnaire should not be applied (use question 39 from the general questionnaire).**

***ALL RESPONDENTS SHOULD ANSWER QUESTION 1.**

**1.** How much money do you spend in a week on sodas you drink? (Do not consider light or diet soda). Include the money that other persons pay on behalf of you.

Less than 10 pesos……………………….…………………………………………………….1

From 10 to 20 pesos…………………….……………………………………………………….2

More than 20 and up to 30 pesos....………………………………………………………………………….3

More than 30 and up to 40 pesos.…………………………………………………………………………….4

More than 40 and up to 50 pesos………..…………..……………………………………………………….5

More than 50 and up to 60 pesos……..………………..…………………………………………………….6

More than 60 and up to 70 pesos…………..………………………………..……………………………….7

More than 70 and up to 80 pesos…..……………………………………..………………………………….8

More than 80 and up to 90 pesos….……………………………………...………………………………...10

More than 90 and up to 100 pesos….…………............……………...……………………………………..11

More than 100 pesos……………………………………………………………………………12

For those who answered **yes** to question 232c (Would you like to reduce your sugar-sweetened beverages intake?), Ask section A .

**Section A**

**2.** Imagine there is a nutrition program that aims to change your soda consumption habits that would reduce in half your current consumption as long you remain in the program. This program does not require you to take pills or any surgery. You are supposed to pay for it, and it is not provided by the government or any other Health institution (IMSS, ISSSTE, PEMEX, SS).

Considering your monthly income and expenditures, are you willing to pay $XXX per month for this intervention to reduce your soda consumption?

|  | $50 | $100 | $200 | $300 | $400 | $500 |
| --- | --- | --- | --- | --- | --- | --- |
| Initial amount |  |  |  |  |  |  |
| Final amount |  |  |  |  |  |  |
| Yes/no to final amount |  |  |  |  |  |  |

**3.** Now think about the health benefits of reducing soda consumption: you could lose weight, reduce risk of diseases such as diabetes, or if you have a disease, you may improve your health condition.

Considering your monthly income and expenditures, are you willing to pay $XXX per month for this intervention to reduce your soda consumption?

|  | $50 | $100 | $200 | $300 | $400 | $500 |
| --- | --- | --- | --- | --- | --- | --- |
| Initial amount |  |  |  |  |  |  |
| Final amount |  |  |  |  |  |  |
| Yes/no to final amount |  |  |  |  |  |  |

**4.** Now consider additional benefits if the money that you pay for this program is used to fund water fountains in the poorest regions of the country and public schools, and treatment of chronic diseases such as diabetes.

Considering your monthly income and expenditures, are you willing to pay $XXX per month for this intervention to reduce your soda intake?

|  | $50 | $100 | $200 | $300 | $400 | $500 |
| --- | --- | --- | --- | --- | --- | --- |
| Initial amount |  |  |  |  |  |  |
| Final amount |  |  |  |  |  |  |
| Yes/no to final amount |  |  |  |  |  |  |

**QUESTIONNAIRE ENDS. THANKS FOR YOUR VALUABLE PARTICIPATION.**

**Section B**

For those who answered **NO** in question 232c (Would you like to reduce your sugar-sweetened beverages intake?), Ask section B .

**2.** We know that you answered in question 232C that you do not have the intention to reduce your soda consumption, but please imagine there is a nutrition program that aims to change your soda consumption habits that would reduce in half your current consumption as long you remain in the program. This program does not require you to take pills or any surgery. You are supposed to pay for it, and it is not provided by the government or any other Health institution (IMSS, ISSSTE, PEMEX, SS).

Considering your monthly income and expenditures, are you willing to pay $XXX per month for this intervention to reduce your soda consumption?

|  | $50 | $100 | $200 | $300 | $400 | $500 |
| --- | --- | --- | --- | --- | --- | --- |
| Initial amount |  |  |  |  |  |  |
| Final amount |  |  |  |  |  |  |
| Yes/no to final amount |  |  |  |  |  |  |

**3.** Now think about the health benefits of reducing soda consumption: you could lose weight, reduce risk of diseases such as diabetes, or if you have a disease, you may improve your health condition.

Considering your monthly income and expenditures, are you willing to pay $XXX per month for this intervention to reduce your soda consumption?

|  | $50 | $100 | $200 | $300 | $400 | $500 |
| --- | --- | --- | --- | --- | --- | --- |
| Initial amount |  |  |  |  |  |  |
| Final amount |  |  |  |  |  |  |
| Yes/no to final amount |  |  |  |  |  |  |

**4.** Now consider additional benefits if the money that you pay for this program is used to fund water fountains in the poorest regions of the country and in public schools, and treatment of chronic diseases such as diabetes.

Considering your monthly income and expenditures, are you willing to pay $XXX per month for this intervention to reduce your soda intake?

|  | $50 | $100 | $200 | $300 | $400 | $500 |
| --- | --- | --- | --- | --- | --- | --- |
| Initial amount |  |  |  |  |  |  |
| Final amount |  |  |  |  |  |  |
| Yes/no to final amount |  |  |  |  |  |  |

**QUESTIONNAIRE ENDS. THANKS FOR YOUR VALUABLE PARTICIPATION.**
