# Supplementary material for: Willingness to pay for an intervention that reduces soda consumption among a sample of middle-class adult Mexicans
Source: PLoS One. 2021 Aug 2;16(8):e0255100. doi: 10.1371/journal.pone.0255100 (PMC8328282; doi:10.1371/journal.pone.0255100)
Supplement: S1 Fig — (DOCX) [file pone.0255100.s002.docx]

**S1 Fig. Example of responses in the bidding process for a sequence of questions when the random start value is 100 pesos.**
