# Supplementary material for: Willingness to pay for an intervention that reduces soda consumption among a sample of middle-class adult Mexicans
Source: PLoS One. 2021 Aug 2;16(8):e0255100. doi: 10.1371/journal.pone.0255100 (PMC8328282; doi:10.1371/journal.pone.0255100)
Supplement: Attachment — Submitted filename: Reviewer report.pdf [file pone.0255100.s003.pdf]

Reviewer report

Mexico has adopted a SSB tax to control the obesity and related chronical diseases. It has been discovered to be significantly decreases the soda consumption. This article contributes to the literature in assessing the willingness to reduce soda consumption and the willingness to pay (WTP) for various interventions that reduces soda consumption. This article focuses on providing knowledges of the benefits and welfare implications of these interventions, including reductions in consumption, health benefits such as reductions in obesity, diabetes and other chronic disease, and using money collected by SSB sales to treatment of chronic diseases and to potable water in the most deprived regions and public schools in Mexico. The author(s) found that people would like to reduce soda consumption, and as more beneficial information is provided, people's WTP increases.

In general, the topic is interesting. This article fills the gap in exploring the relation between WTP for soda drinks and interventions—health benefits and social benefits.

Major comments

1. There are 1241 obs. in the third wave of HWSC, however the analytical sample is 470 in this study. Why did the rest of the individuals not participate in the WTP study? Is there any sample selection criterion? Are **sample weights** used in the estimation?
2. In page 8, "We also assessed whether the subsample of the WTP study was representative of the HWSC by testing for statistical differences in the co-variables included in the analysis between our analytical sample and all participants of the cohort in the third wave." Can you provide the details? Whether the difference is significant or not for each explanatory variable?

3. Although by reading the whole manuscript, we are able to figure out that interval regression is applied in the empirical estimation. I strongly suggest the author(s) to provide detailed description on the methodology used in the manuscript.
4. Sample size is too small (471). Robustness check should be conducted. Proper empirical approach should be used to address the data limitation, i.e., bootstrap.
5. Whether the estimation (Table 3) is clustered by household or else, there is no specification on it.
6. I think authors should focus more on potential policy implications regarding to various responses to tax revenue recycling. For example, if individuals are discovered to be more altruistic –using the collected money to help the poor, what kind of policy should the government adopt? Or else, individuals are revealed to be more conscious of heath benefits, what kind of scenario should be used?
