# Supplementary material for: Willingness to pay for an intervention that reduces soda consumption among a sample of middle-class adult Mexicans
Source: PLoS One. 2021 Aug 2;16(8):e0255100. doi: 10.1371/journal.pone.0255100 (PMC8328282; doi:10.1371/journal.pone.0255100)
Supplement: Attachment — Submitted filename: CoverLetter PONE-D-20-34196.pdf [file pone.0255100.s005.pdf]

Referee Report Cover Letter
Manuscript PONE-D-20-34196

February 13th, 2021
Professor Shihe Fu
Editor, PLOS ONE

Dear Professor Fu,

Thank you for inviting me to review the manuscript titled "Willingness to pay for an intervention that reduces soda consumption".

I recommend that the manuscript be rejected. The authors failed to clarify whether there would be changes the choice set aside from a change in preference in the hypothetical scenario in the questionnaire when they elicited willingness to pay for an intervention of soda reduction. As a result, the analysis does not accomplish what it set out to do. This conceptual flaw convinced me that the paper does not meet the standard required for publishing in PLOS ONE.

I explained my assessment in detail in the body of referee report. Please let me know if you have questions.

Sincerely,

Zhe Yang
Assistant Professor
Institute of Economic and Social Research
zheyang@ucdavis.edu
