# Supplementary material for: Willingness to pay for an intervention that reduces soda consumption among a sample of middle-class adult Mexicans
Source: PLoS One. 2021 Aug 2;16(8):e0255100. doi: 10.1371/journal.pone.0255100 (PMC8328282; doi:10.1371/journal.pone.0255100)
Supplement: Attachment — Submitted filename: WTP_Responses to reviewers R2_21Jun21.docx [file pone.0255100.s007.docx]

Reviewers' comments:

Reviewer #1: The author has addressed most of my questions. Although sample size and its representativeness still bothers, the use of bootstrap method and appropriate empirical approaches could to some extent address it.

**Response: Thank you.**

**Reviewer #2**: Question 2 in the survey asks ``Imagine there is a nutrition program that aims to change your soda consumption habits that would reduce in half your current consumption as long you remain in the program. This program does not require you to take pills or any surgery. You are supposed to pay for it, and it is not provided by the government or any other Health institution." The wording implies that the method through which the hypothetical intervention would reduce soda consumption is a change in preferences without restricting the choice set, which differs from what actual interventions usually do, such as a tax on soda consumption and regulation of the beverage in schools. In the latter cases, there might be large reduction in consumer's maximized utility caused by the shrinking of the choice set. Because they are not informed about these potential losses, the respondents will considerably overestimate their WTP.

Response: We agree with the reviewer. We added the following in the discussion section.
“The WTP reflects the preference to quit or to reduce soda consumption. We acknowledge that we cannot translate this social value as if we would implement an intervention that restricts the choice set.”

Response to authors’ response: The word “quit” plays a key role in clarifying that the WTP is for removing soda addiction instead of restricting soda consumption. The ambiguity between the two was central in the issue in my previous comments. It might be helpful to solve the issue if you could also implement this clarification to the ambiguous “willingness to reduces SSB consumption” in the abstract and many other places in the manuscript.

**Response: We agree with the reviewer. We are not measuring willingness to reduce SSB consumption. The questionnaire asks individuals for their intention to reduce soda consumption. We replaced “willingness to reduce soda consumption” to “intention to reduce soda consumption” all over the manuscript.**

**We also agree that we are not restricting the choice set.**

**We added the following:**

“The WTP reflects the preference to reduce soda consumption. We acknowledge that we cannot translate this social value as if we would implement an intervention that restricts the choice set. **For those who had the intention to reduce soda consumption, the nutrition program aligns their preferences to their habits. For those who did not have the intention to reduce soda consumption, the program changes their preferences. In both cases, the program does not restrict the choice set.”**

Minor Comments:
[1] It is not clear if sample weights are used to infer the WTP of the population. Weighting could be important when the demographic composition in the sample is different from that in the population to the extent that 75% of the sample are female.

Response: There are no sample weights in the data set. As acknowledged in the discussion section, the cohort is not representative of the population as it is for the Health Workers Cohort Study. However, we tested for statistical differences in basic characteristics between our analytic sample and the third wave of the Health Workers Cohort Study and we did not find significant differences.

Response to authors’ response: I have no further issue with sample weights.

**Response: Thank you.**

[2] One of the independent variables in the regression analysis is the 0-1 variable ``would like to reduce soda." It would be a bad control if it is an outcome rather than a cause of the WTP to reduce soda.

Response: We understand the concern. We ran the model without the variable and the results are very similar. We also ran a probit model with the “would like to reduce soda” as the dependent variable and WTP as an independent variable, adjusting for the same variables and we did not find a significant association. We added the following in the discussion section:
“We acknowledge a potential reverse causality if willingness to reduce soda consumption, that we use as an independent variable, causes WTP. We followed two approaches. We first ran the interval regression excluding willingness to reduce soda consumption and found that the results are very similar. We also ran a probit model with a binary variable of willingness to reduce soda consumption as the dependent variable, willingness to pay as independent variable, and the other covariates but the coefficient for WTP was not statistically significant”.

Response to authors’ response: Does “Would like to reduce soda=0” imply “WTP=0” in theory? How does isolating the effect of “WTP>0” on WTP help us understand WTP?

**Response: We are not assuming that those who did not have an intention to reduce soda had a willingness to pay equal to zero since, as mentioned above, the program can change their preferences. Actually, their WTP is greater than zero, lower than those who had the intention to reduce soda but not zero. We added in table 2, the average WTP by intention to reduce soda consumption by scenario.**

**We added the following in the methods section:**

“We first described **the average WTP by intention to reduce soda consumption by scenario** and the unadjusted distribution of the WTP for the three scenarios: 1) cut in soda consumption, 2) cut in soda consumption + explicitly accounting for individual health benefits, 3) adding social benefits.”

**We added the following in the results section (description of table 2):**

**“**Table 2 shows **the average WTP by intention to reduce soda consumption and** the distribution of the WTP intervals by scenario**. Those who had the intention to reduce soda consumption had a higher WTP compared to those who had not intention to reduce soda consumption but their WTP. The average WTP for the soda consumption reduction scenario was 244.9 pesos for those with the intention to reduce soda consumption, 280.5 for the scenario that adds health benefits and 302.6 for the scenario that add social benefits. For those with no intention to reduce soda consumption the average WTP was 162.5, 212.0 and 264.5, respectively.”**
