# Supplementary material for: Willingness to pay for an intervention that reduces soda consumption among a sample of middle-class adult Mexicans
Source: PLoS One. 2021 Aug 2;16(8):e0255100. doi: 10.1371/journal.pone.0255100 (PMC8328282; doi:10.1371/journal.pone.0255100)
Supplement: Attachment — Submitted filename: Referee Report PONE-D-20-34196.pdf [file pone.0255100.s004.pdf]

Referee Report on “Willingness to pay for an intervention that reduces soda consumption among a sample of middle-class adult Mexicans”

M. A. Colchero, Carlos M Guerrero-Lpez, Tonatiuh Barrientos-Gutirrez, Jorge Salmern and Sergiio Bautista-Arredondo

Manuscript Number: PONE-D-20-34196

In this paper, the authors assess the willingness to pay (WTP) for an intervention that would reduces soda consumption by half in Mexico. Using contingent valuation methods in a sample of 471 Mexican adults from a cohort study, they find that high soda consumption, willingness to reduce soda consumption , higher household income, and additional information about the health and social benefits of reducing soda consumption are associated with higher WTP. They also find that the WTP, as a proportion of income, is higher for the lowest income level.

This paper contributes to the policy debate on the social value of a reduction in soda consumption in Mexico by directly eliciting the population's WTP to reduce soda consumption. However, the contribution is severely compromised by the way the intervention was interpreted in the questionnaire.

Question 2 in the survey asks “Imagine there is a nutrition program that aims to change your soda consumption habits that would reduce in half your current consumption as long you remain in the program. This program does not require you to take pills or any surgery. You are supposed to pay for it, and it is not provided by the government or any other

Health institution.” The wording implies that the method through which the hypothetical intervention would reduce soda consumption is a change in preferences without restricting the choice set, which differs from what actual interventions usually do, such as a tax on soda consumption and regulation of the beverage in schools. In the latter cases, there might be large reduction in consumer’s maximized utility caused by the shrinking of the choice set. Because they are not informed about these potential losses, the respondents will considerably overestimate their WTP.

Minor Comments

It is not clear if sample weights are used to infer the WTP of the population. Weighting could be important when the demographic composition in the sample is different from that in the population to the extent that 75% of the sample are female.

One of the independent variables in the regression analysis is the 0-1 variable “would like to reduce soda.” It would be a bad control if it is an outcome rather than a cause of the WTP to reduce soda.
